# Safety, immunogenicity, and baseline immune correlates of vaccine JNJ-0535 in participants with or without CHB

**DOI:** 10.1038/s41541-025-01364-x

**Published:** 2026-02-05

**Authors:** Simon Verheijden, Nádia Conceição-Neto, Stefan Bourgeois, Céline Vandamme, Ewoud de Troyer, Evangelos Kanoulas, Dries De Maeyer, Marjolein Crabbe, Elli Makariadou, Leen Slaets, Bart Fevery, Pieter Van Remoortere, Michael Biermer, Patrick T. F. Kennedy, An De Creus

**Affiliations:** 1 Johnson & Johnson, Beerse, Belgium; 2 ZNA Jan Palfijn, CPU, Antwerp, Belgium; 3 Johnson & Johnson, Titusville, NJ USA; 4 Blizard Institute, Barts and The London School of Medicine and Dentistry, Queen Mary University of London (QMUL), London, UK

**Keywords:** Biomarkers, Medical research

## Abstract

The role of T-cell-mediated immune responses is recognized as pivotal in achieving functional cure in chronic hepatitis B (CHB) patients. We aimed to assess safety and T-cell responses induced by JNJ-6430535 (JNJ-0535); a hepatitis B virus (HBV)-specific therapeutic DNA vaccine administered via electroporation-mediated intramuscular injection. JNJ-0535 comprises 2 plasmids, encoding HBV core and polymerase (pol) proteins, respectively. We describe the safety, tolerability, and immunogenicity results of JNJ-0535 from an open-label, single arm phase 1 study in healthy volunteers (HVs) (64300535HPB1003, NTC04736147) and a randomized, placebo controlled phase 1 study in CHB patients (64300535HPB1001, NTC03463369). HBV-specific T-cell responses were evaluated using enzyme-linked immunospot (ELISpot) and intracellular cytokine staining (ICS). We performed baseline single-cell RNA sequencing (scRNA-seq) to explore immune correlates associated with vaccine response in HVs, and baseline serum proteomics (Olink Explore^®^ 3072) to explore differences in soluble immune markers between responders and non-responders in both HVs and CHB patients. JNJ-0535 was safe and well tolerated in both HVs and CHB patients. Compared to CHB patients, HVs showed a higher proportion of participants with vaccine-induced HBV-specific T-cell responses (92% versus 50%), a greater increase from baseline (24× [interquartile range=40×;9×] versus 4.8× [interquartile range=5×;6×]) and a broader response in terms of number of antigens. Serum proteomics revealed few differential circulating host biomarkers between CHB and HVs, but these could not be linked to differences in immunogenicity. In addition, whole-blood scRNA-seq was performed in HVs to explore differences between participants with a strong vaccine response and those with low or no response. Our research showed a decrease in vaccine-induced responses in CHB patients compared to HVs and may provide preliminary insights into immune-related biomarkers that could influence vaccine immunogenicity but require further confirmation in future larger studies. **trial numbers:** 64300535HPB1001 (NCT03463369) First posted: 18. April 2018; ULR: https://clinicaltrials.gov/study/NCT03463369?term=JNJ-64300535&rank=2 and 64300535HPB1003 (NCT04736147) First posted: 03. Feb. 2021; URL: https://clinicaltrials.gov/study/NCT04736147?term=JNJ-64300535&rank=1.

## Introduction

An estimated 296 million people worldwide are chronically infected with HBV. Chronic hepatitis B (CHB) is associated with increased risk of liver disease progression to liver cirrhosis, hepatic failure, and/or hepatocellular carcinoma (HCC)^1^. Currently available treatments are suppressing viral replication either directly (i.e., nucleos(t)ide analogues [NAs]) or with an additional immune-stimulatory effect (i.e., pegylated interferon-alpha [pegIFN-α]). The clinical goal for patients with CHB is to achieve a ‘functional cure’, defined as undetectable serum levels for hepatitis B surface antigen (HBsAg) and HBV DNA that is sustained for ≥24 weeks beyond the end of treatment^2^. Functional cure is only rarely achieved with currently available treatments highlighting the need for novel therapeutic strategies. Although preventive HBV vaccines based on HBsAg are available since 1983 and highly effective in non-infected individuals, they are not effective in patients who are already infected.

Although the mechanisms for viral persistence and chronicity are not yet fully understood, it is widely accepted that immunologic mechanisms are involved in several aspects of the pathogenesis, natural course, destruction of infected cells, and control of viral replication. More than 90% of adults who become infected with HBV show a natural resolution of infection. In these patients, HBV-specific CD4 and CD8 T-cell responses can be detected ex vivo^3^. However, in CHB these responses are less frequent and functionally impaired, leading to the limited ability of the immune system to eliminate the virus^4^. These findings suggest that it may be possible to achieve functional cure for HBV by restoring HBV-specific immune responses that resemble those observed in individuals with natural resolution of infection. Therapeutic vaccination with HBV antigens could provide a novel approach to induce either new or boost existing HBV-specific T-cell responses.

A therapeutic vaccine should preferentially induce a strong CD4 T helper (Th)1 and cytotoxic CD8 cellular response, as CD4 Th1 cells play a key role in the clearance of HBV by enhancing the effector responses of the virus-specific CD8 T cells^5,6^. Until now, attempts to use therapeutic vaccination for CHB to achieve functional cure have not been successful^7^. This lack of success might be due to 1) the vaccines used not being sufficiently immunogenic and 2) CHB patients having been exposed to HBV antigens for a longer period, resulting in exhaustion and potentially depletion of HBV-specific T cells, which may attenuate responses to the vaccine. The HBV-specific therapeutic vaccine JNJ-64300535 (JNJ-0535) used in these studies is a DNA vaccine that consists of 2 non-infectious DNA plasmids, directing T-cell responses to the core and polymerase (pol) proteins of HBV, whose coding sequences are highly conserved between genotypes. JNJ-0535 is administered via electroporation-mediated intramuscular (i.m.) injection to enhance the immunogenicity of the vaccine^8^. We have previously shown that HBV core and pol proteins delivered i.m. via electroporation induce robust HBV-specific immune responses in healthy and adeno-associated hepatitis B virus (AAV-HBV)-infected mice as well as in healthy non-human primates^9^.

Herein we describe the safety and tolerability of JNJ-0535 and compare HBV-specific T-cell responses induced and expanded by the maximal feasible dose of 6 mg of JNJ-0535 in both healthy volunteer (HV) and CHB patients. To achieve this, we employed a multifaceted approach, combining the interferon (IFN)-γ enzyme-linked immunoSpot (ELISpot) and intracellular cytokine staining (ICS) assays to determine the functionality and magnitude of HBV-specific T-cell responses. Furthermore, serum protein profiling (Olink^®^ Explore 3072) and whole-blood single-cell RNA sequencing (scRNA-seq) was performed at baseline to explore immunological signatures associated with vaccine response.

## Results

### JNJ-0535 was safe and well tolerated in both CHB patients and HVs

A summary of TEAEs from both studies by treatment arm is provided in Supplementary Table 1. In the CHB study, serious AEs were reported in 1 participant on 1 mg (i.e., tuberculosis, multiple injuries, reported as Grade 4, and 2 events of intestinal obstruction of which 1 was reported as Grade 3), in 1 participant on 6 mg (i.e., pneumonia), and in 1 participant on placebo (i.e., miscarriage of partner, reported as Grade 3). No deaths or TEAEs leading to discontinuation of study treatment were reported. Most TEAEs were Grade 1 in severity and none of Grade 3, Grade 4, or serious AEs were considered related to the study treatment by the Investigator. The most frequently reported TEAEs were general disorders and administration site conditions (reported in 61% of participants on JNJ-0535 and 29% on placebo), and infections and infestations (reported in 57% of participants on JNJ-0535 and 42.9% on placebo). No dose-dependent trends were observed. Most related TEAEs were considered related to both JNJ-0535/placebo and the electroporation device and were general disorders and administration site conditions. In all participants, these events were Grade 1 in severity, except for 1 event of Grade 2 injection site pain in a participant who received 6 mg.

For the HV cohort, no serious AEs were reported. For 1 (7.1%) participant, a TEAE leading to discontinuation of JNJ-0535 was reported on Day 1 after the first vaccination, i.e., non-serious, Grade 1 TEAE syncope, related to JNJ-0535 and the electroporation device.

### No observed effect of JNJ-0535 on virological and serological parameters in CHB patients

Levels (quantitative) of HBV DNA, HBV RNA, HBcrAg, HBsAg, and anti-HBs, as well as the status (qualitative) of HBeAg and anti-HBe were monitored at regular time points during the vaccination and follow-up period. All participants were HBeAg negative and virologically suppressed (HBV DNA < 20 IU/mL) at baseline. All participants remained HBeAg negative throughout the study and one participant who received 1 mg JNJ-0535 experienced a HBV DNA virological breakthrough while on NA treatment during FU, accompanied by an anti-HBe seroconversion from negative to positive. No relevant changes from baseline were observed in any of the dose groups for any of the other virological and serological parameters HBV RNA, HBsAg, anti-HBs and HBcrAg (data not shown). No participants experienced HBsAg seroconversion in this study.

### JNJ-0535 induced stronger HBV-specific T-cell responses in HVs compared to CHB patients

First-in-human study 64300535HPB1001 evaluated total doses of 0.25 mg (n = 6), 1 mg (n = 7), or 6 mg (n = 10) JNJ-0535 or placebo (n = 7) in CHB patients who were virologically suppressed, HBeAg-negative, and on stable NA therapy. In CHB patients, a ≥ 3-fold increase in ELISpot responses over baseline to core and/or pol was observed in 2 of 7 patients (28.6%) at 1 mg and 5 of 10 patients (50%) at 6 mg JNJ-0535. Study 64300535HPB1003 evaluated only the highest dose of 6 mg JNJ-0535 in HVs (n = 12).

**Fig. 1:**
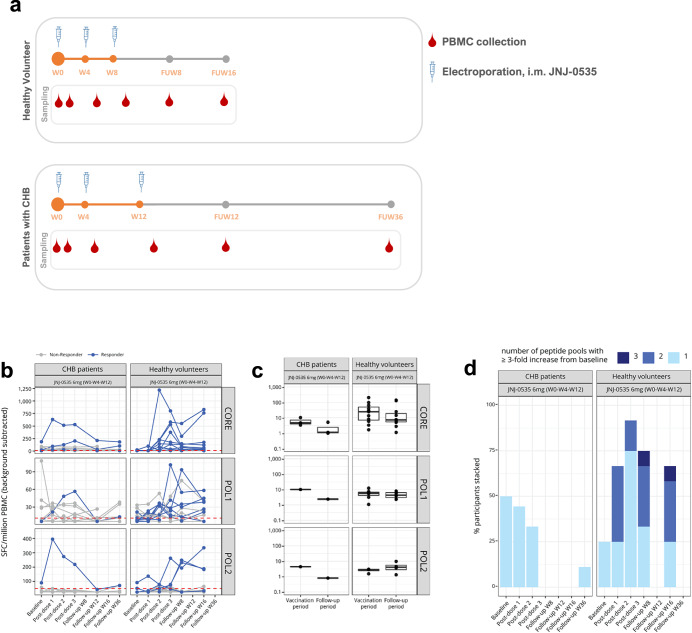
HBV-specific T cell responses in CHB patients compared to HVs after vaccination with JNJ-0535. PBMCs isolated from HVs and CHB patients were overnight stimulated with peptides pools specific for core, pol1, and pol2. HBV peptide-specific responses were measured with IFN-γ ELISpot (SFC/10^6^ PBMCs). All values are mock-subtracted and values below PT/2 were imputed by PT/2. **a** Scheme of vaccination. HV and CHB patients were i.m. immunized with 6 mg of JNJ-0535 at Day 1 (W0), Week 4 (W4), and Week 8 (W8) or W0 W 4 and W12, respectively. PBMC were collected, 10 or 14 days post each immunization and during follow-up (FU). **b** Magnitude and durability of T-cell responses to core, pol1, and pol2 at each timepoint after vaccination. Dark blue lines, JNJ-0535 ELISpot responders ( ≥ 3-fold increase from baseline during vaccination period); grey lines, non-vaccine responders. Dotted red line indicates PT per peptide pool: PT core = 11.9 SFC/10^6^ PBMCs; PT pol1 = 11.3 SFC/10^6^ PBMCs; PT pol2 = 50.9 SFC/10^6^ PBMCs. **c** Median maximum fold changes from baseline values for each HBV-specific peptide pool during vaccination and follow-up period. **d** Percentage of participants responding to 1, 2, or 3 HBV-specific peptide pools at each timepoint after vaccination. CHB chronic hepatitis B, ELISpot enzyme-linked immunospot, HV healthy volunteer, IFN interferon, JNJ-0535 JNJ-64300535, PBMC peripheral blood mononuclear cell, pol polymerase, PT positivity threshold, SFC spot-forming cells, W week.

To determine if the immune-suppressive effects resulting from a long-term HBV infection affected JNJ-0535’s ability to stimulate HBV-specific T-cell responses, we examined the T-cell responses in HV and CHB patients before and after vaccination with the highest dose, i.e., 6 mg of JNJ-0535. PBMCs collected at baseline, at 10 or 14 days after each of the 3 vaccinations, and at multiple timepoints during FU were evaluated for T-cell responses against core and pol using ex vivo IFN-γ ELISpot and ICS (Fig. 1a).

JNJ-0535 vaccination induced a higher proportion of responders in HVs compared to CHB patients. In HVs using IFN-γ ELISpot, 11 of 12 participants (91.7%) showed an increase ( ≥ 3-fold from baseline) in detectable HBV-specific T cells to at least 1 vaccine antigen during the vaccination period when receiving 3 doses of 6 mg (Fig. 1b and Table 1). On the other hand, in participants with CHB, only 5 of 10 patients (50.0%) responded to at least 1 of the vaccine antigens after receiving 3 doses of 6 mg. During the FU period, the HBV-specific T-cell responses induced by the vaccine were mostly maintained in HVs (9 of 12 participants, 75%), while in participants with CHB, only 1 of 10 patients (10%) had induced or expanded responses, which returned to baseline levels. During the vaccination period, 10 of the HVs responded to core, 6 to pol1, and 1 to pol2, while during the FU period, the corresponding numbers were 9, 5, and 3. Three CHB patients responded to core, 1 to pol1, and 1 to pol2 during the vaccination period, while during the FU only 1 participant with CHB still responded to core and responses to pol were lost.

**Table 1:** Overall and peptide-pool-specific vaccine responders in CHB patients compared to HVs during the vaccination and follow-up period

	CHB patients 6 mg JNJ-0535 W0-W4-W12	Healthy Volunteers 6 mg JNJ-0535 W0-W4-W8
Overall responders	n/N%	n/N%
Vaccination period		
At least 1 peptide pool	5/10 (50.0%)	11/12 (91.7%)
Follow-up period	FU W12, FU W36	FU W8, FU W16
At least 1 peptide pool	1/10 (10.0%)	9/12 (75.0%)
Peptide pool-specific responders	n/N%	n/N%
Vaccination period		
Core	3/10 (30.0%)	10/12 (83.3%)
Pol1	1/10 (10.0%)	6/12 (50.0%)
Pol2	1/10 (10.0%)	1/12 (8.3%)
Follow-up period		
Core	1/10 (10.0%)	9/12 (75.0%)
Pol1	0/10	5/12 (41.7%)
Pol2	0/10	3/12 (25.0%)

Mock-subtracted values are used in the analysis and mock-subtracted values < PT/2 are imputed by PT/2.

*CHB* chronic hepatitis B, *FU* follow-up, *HV* healthy volunteer, *JNJ-0535* JNJ-64300535, *pol* polymerase, *W* week.

In addition, JNJ-0535 vaccination induced also a higher median maximum fold-change in core-specific T-cell levels from baseline in HVs (i.e., median 24.2-fold increase) compared to CHB patients (i.e., 4.8-fold increase) during the vaccination period (Fig. 1c and Table 2). However, for participants who responded to pol, less differences were observed in the median maximum fold-change during vaccination between HVs and CHB patients.

**Table 2:** Maximum fold-change from baseline after vaccination in CHB patients and HVs during vaccination and follow-up period, and per antigen

	CHB patients 6 mg JNJ-0535 W0-W4-W12	Healthy Volunteers 6 mg JNJ-0535 W0-W4-W8
Maximum fold-change	N Median (range)	N Median (range)
Vaccination period		
Core	3 4.8 (3.4–10.2)	3 24.4 (1.7–204.8)
Pol1	1 10.0	7 5.6 (1.1–12.3)
Pol2	1 4.4	4 2.9 (1.5–3.0)
Follow-up period	FU W12, FU W36	FU W12, FU W36
Core	3 1.1 (1.0–5.2)	11 7.2 (1.1–139.2)
Pol1	1 2.4	7 4.3 (2.3–8.0)
Pol2	1 0.8	4 4.1 (1.3–9.8)

Descriptive statistics of the maximum fold change per participant during each period per antigen are shown. Median and range of fold change is calculated only for N = total number of participants (with a response to the specific peptide pool. Mock-subtracted values are used in the analysis and mock-subtracted values < PT/2 are imputed by PT/2.

*CHB* chronic hepatitis B, *FU* follow-up, *HV* healthy volunteer, *JNJ-0535* JNJ-64300535, *pol* polymerase, *W* week.

Moreover, JNJ-0535 vaccination resulted in higher breadth of vaccine-induced T-cell responses in HVs compared to CHB patients. Of the HVs, 50% (6 of 12) showed a response to both core and pol (2 peptide pools: core and pol1 or pol2) during the vaccination period (Fig. 1d), and the breadth of responses induced by JNJ-0535 vaccination was maintained over time as 50% of the HVs still responded to both core and pol (2 peptide pools: core and pol1 or pol2) during FU, with 17% of the participants showing a response to all 3 peptide pools (core, pol1, and pol2). In contrast, 0% of CHB patients (0 of 10) responded to both core and pol either during vaccination or during FU.

No trend was observed with any viral parameters and vaccine responders or non-responders in CHB patient, albeit data was limited. HBsAg and HBcrAg titers did not differ between baseline and any timepoint post vaccination. Anti-HBs, which was undetectable at baseline, remained undetectable at end of study. (data not shown).

### No trend was observed in terms of baseline characteristics and immunogenicity

In accordance with the study design, the HV and CHB cohort (6 mg dose) were not matched for demographic characteristics. The HV cohort consisted predominantly of white males, and a median age of 45.5 years. In contrast, the CHB cohort was more heterogeneous, comprising six males and four females, with participants of both Asian and Black ethnicities, and a median age of 49.5 years (Table 3). Despite these differences, no apparent trend was observed between age and immunogenicity within either cohort. Additionally, analysis of other baseline characteristics and immunogenicity data in the CHB cohort did not reveal any significant trends. It is important to note that the sample size in both the HV and CHB group were small, which limits definitive conclusions. Nonetheless, these observations suggest that demographic variables such as age and other baseline characteristics might not substantially influence the immunogenic responses observed in HV or CHB patients.

**Table 3:** Baseline characteristics and ELISpot vaccine responders

	Sex	Age	Race	HBV DNA (log10 IU/mL)	HBcrAg (log10 IU/mL)	Anti-Hbe	HbeAg	Anti-HBs (IU/L)	HBsAg (log10 IU/mL)	HBV RNA (log10 copies/mL)	CHB diagnosis (years before screening)	Liver Stiffness (pKa)	ELISPOT responder
CHB patient	M	38	Black or African American	<LLOQ	3.8	POS	NEG	2.5	3.8	2.3	8	5.6	No
	M	38	–	TND	<LLOQ	POS	NEG	2.5	3.6	0.7	–	5.9	No
	F	52	–	TND	<LLOQ	POS	NEG	2.5	2.9	0.7	26	1.0	No
	M	55	White	TND	3.1	NEG	NEG	2.5	2.8	2.1	22	4.4	No
	M	47	White	<LLOQ	<LLOQ	POS	NEG	2.5	3.6	0.7	14	0.7	No
	F	53	White	TND	<LLOQ	POS	NEG	2.5	3.4	0.7	35	3.3	Yes
	F	53	White	TND	3	POS	NEG	2.5	3.6	0.7	28	1.2	Yes
	M	41	Asian	<LLOQ	<LLOQ	POS	NEG	2.5	2.6	0.7	32	3.5	Yes
	F	62	White	TND	<LLOQ	POS	NEG	2.5	3.7	0.7	22	7.3	Yes
	M	39	Asian	TND	<LLOQ	POS	NEG	2.5	2.3	0.7	34	5.0	Yes
HV	M	46	White	na	na	na	na	na	na	na	na	na	No
	M	35	White	na	na	na	na	na	na	na	na	na	Yes
	M	32	White	na	na	na	na	na	na	na	na	na	Yes
	M	26	White	na	na	na	na	na	na	na	na	na	Yes
	M	24	White	na	na	na	na	na	na	na	na	na	Yes
	M	53	White	na	na	na	na	na	na	na	na	na	Yes
	M	51	White	na	na	na	na	na	na	na	na	na	Yes
	M	31	White	na	na	na	na	na	na	na	na	na	Yes
	M	52	White	na	na	na	na	na	na	na	na	na	Yes
	M	45	White	na	na	na	na	na	na	na	na	na	Yes
	M	53	White	na	na	na	na	na	na	na	na	na	Yes
	M	46	White	na	na	na	na	na	na	na	na	na	Yes

*anti-HBe* anti-HBV e-antigen, *anti-HBs* anti-HBV surface-antigen, *CHB* chronic hepatitis B, *HV* healthy volunteer, *HBcrAg* HBV core-related antigen, *LLO* lower limit of quantification, *TND* target not detected.

### JNJ-0535 vaccination induced polyfunctional core-specific CD4 and CD8 T cells in HVs

**Fig. 2:**
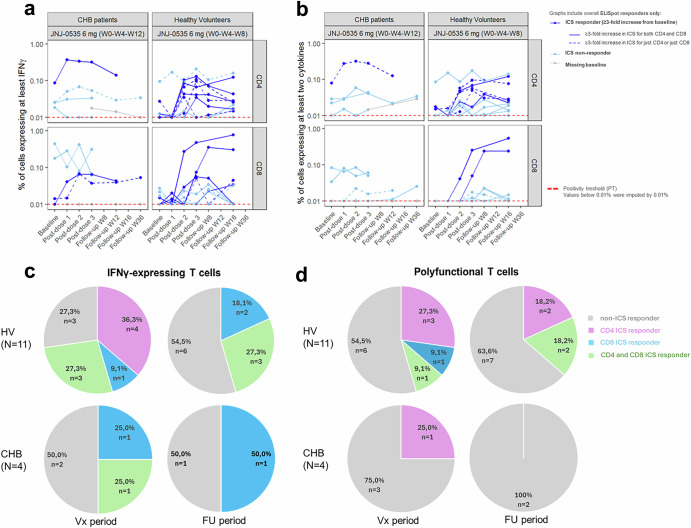
IFN-γ-secreting and polyfunctional core-specific T cells in CHB patients compared to HVs after vaccination with JNJ-0535. PBMCs isolated from HVs and CHB patients were overnight stimulated with peptide pools specific for core. CD4 and CD8 core-specific T-cell responses were measured using ICS. All values are mock subtracted and values < 0.01% are imputed. **a** Percentage of CD4 (top) and CD8 (bottom) core-specific T cells secreting at least IFN-γ. **b** Percentage of polyfunctional CD4 (top) and polyfunctional CD8 (bottom) core-specific T cells expressing at least 2 markers out of IFN-γ, TNF-γ, IL-2, or CD40L. Dark blue lines, JNJ-0535 ICS responders ( ≥ 3-fold increase from baseline during the vaccination period); light blue lines, non-ICS responders. The dotted red line indicates the PTs specific for core-specific CD4 and CD8 IFN-γ secreting or polyfunctional T cells. Pie charts indicating percentage of **c** IFN-γ-expressing and **d** polyfunctional CD4 and/or CD8 core-specific T cells in HV (top) and CHB patients (bottom) during the vaccination (Vx) and FU period. Grey indicates non-ICS responders, purple indicates CD4 ICS responders only, light blue indicates CD8 ICS responders only and green indicates both CD4 and CD8 ICS responders. Participants with missing ICS response (Vx Period: n = 1, CHB FU period: n = 3) were excluded. CD cluster of differentiation, CHB chronic hepatitis B, ELISpot enzyme-linked immunospot, FU Follow-up, HV healthy volunteer, ICS intracellular staining, IFN interferon, JNJ-0535 JNJ-64300535, PBMC peripheral blood mononuclear cell, PT positivity threshold, TNF tumor necrosis factor, Vx Vaccination, W week.

During the vaccination period, JNJ-0535 induced increased frequencies of IFN-γ core-specific CD4 T cells compared to baseline in 64% of HV ELISpot responders (7 of 11) (Fig. 2a, c). Among these responders, in 5 of the 7 HVs, vaccine-induced core-specific CD4 T cells were also polyfunctional, secreting more than 2 cytokines (IFN-γ, TNF-α, or IL-2) and showing a ≥ 3-fold increase from baseline (Fig 2b, d). Additionally, 4 of the 7 HVs showed a ≥ 3-fold increase in IFN-γ CD8 core-specific T cells post vaccination, with 2 of these also showing polyfunctionality (Fig 2 a-d). One additional HV showed an increase in IFN-γ CD8 core-specific T cells during vaccination, without polyfunctionality. Most of these IFN-γ secreting and/or polyfunctional CD4 and CD8 core-specific T-cell responses were still present 16 weeks after the last vaccination. Vaccine responders who only showed IFN-γ or polyfunctional CD8 core-specific T cells, without any CD4 core-specific T cells responses, could no longer be detected during FU.

In contrast with these findings, JNJ-0535 vaccination did not elicit an increase or enhancement in polyfunctional core-specific CD8 T cells after JNJ-0535 vaccination in CHB patients (n = 4), including those with detectable core-specific CD8 T cells at baseline (n = 2) (Fig. 2b, d). In 1 of 4 CHB ELISpot responders (N = 5 but one patient with missing baseline), an increase from baseline in both IFN-γ core-specific CD4 and CD8 T cells was observed. Only the core-specific CD4 T cells were polyfunctional, showing a ≥ 3-fold increase from baseline during the vaccination period but returned to baseline levels at 12 weeks after the last vaccination (Fig. 2a, c). This CHB patient already had a higher frequency of polyfunctional core-specific CD4 T cells detectable at baseline prior to vaccination. In addition, one CHB patient showed an increase only in IFN-γ core-specific CD8 T cells during vaccination that was maintained during the FU period, these cells were not polyfunctional (Fig. 2a–d).

### Baseline serum protein profiling comparing HV versus CHB and ELISPOT response groups

**Fig. 3:**
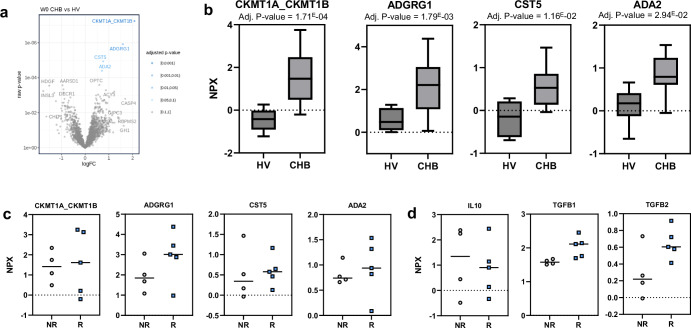
Olink Explore^®^ 3072 baseline serum proteomics comparing CHB patients and HVs. a Volcano plot showing differential protein expression at baseline comparing CHB patients and HVs. **b** Box plots of significantly differentially expressed proteins (adjusted p-value ≤ 0.05) between CHB and HVs. **c** Distribution of expression of significant differential proteins across vaccine responders and non-responders in CHB. **d** Expression of immune-suppressive cytokines in vaccine responders and non-responders in CHB. Adj. adjusted, CHB chronic hepatitis B, HV healthy volunteer, NPX normalized protein expression, W week.

To explore a potential association of baseline serum protein immune signatures with vaccine-induced T-cell responses, 2943 proteins were measured using Olink^®^ Explore 3072 analysis in pre-vaccination serum samples from both HVs and CHB patients. Differential protein analysis identified increased expression of *CKMT1A_CKMT1B*, *ADGRG1*, *CST5*, and *ADA2* in CHB patients versus HVs (adjusted p-value ≤ 0.05) (Fig. 3a, b). To explore if the differential abundance of these proteins could explain the difference in vaccine response rate, the abundance between ELISPOT responders (R) and ELISPOT non-responders (NR) was explored. None of the proteins were different between R and NR in the CHB cohort (Fig. 3c). Furthermore, none of the typical immune-suppressive cytokines (*IL10, TGFB1, TGFB2*) showed lower expression in R vs NR (Fig. 3d).

### Low naïve to effector/memory T cell ratio in HV not responding to JNJ-0535

**Fig. 4:**
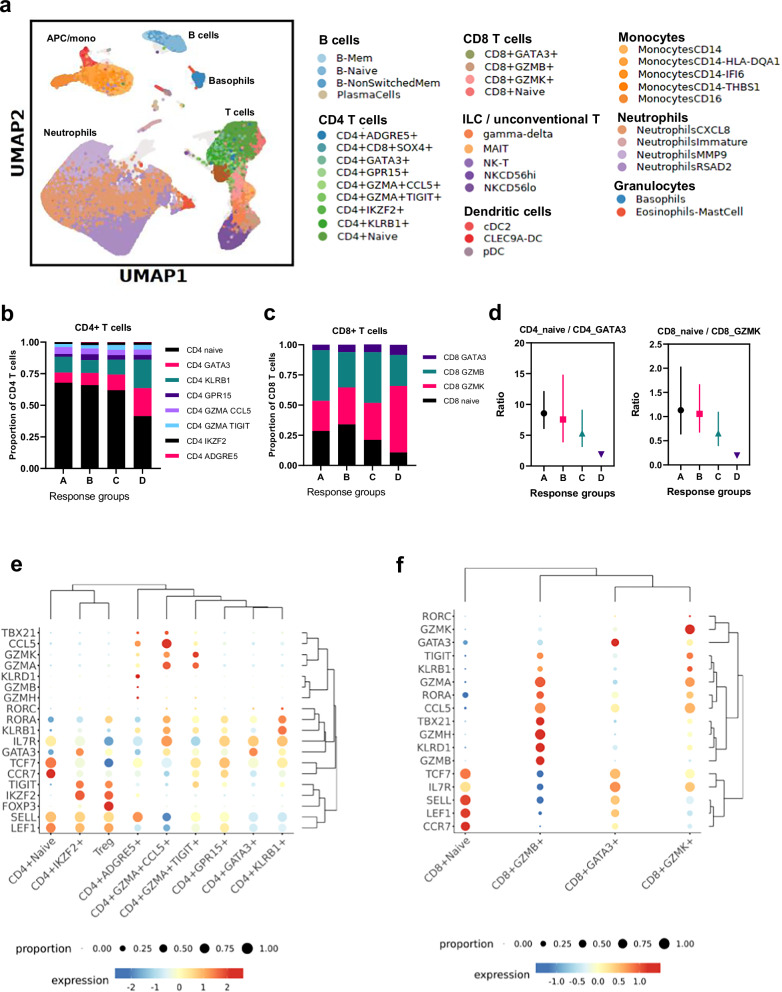
Lower naïve-to-effector memory T cell ratio in HV vaccine non-responder. **a** UMAP of all immune cells characterized by BD Rhapsody® single cell RNA-sequencing. **b** Distribution of CD4 T cell subsets across the different response groups in HVs. **c** Distribution of CD8 T cell subsets across the different response groups in HVs. **d** Ratio of CD4 naïve to CD4 effector memory (CD4_GATA3) (left) and CD8 naïve to CD8 effector memory (CD8_GZMK) (right) across the different response groups in HVs. **e** Expression of naïve, effector, memory, cytotoxic and regulatory markers across different CD4 T cell populations. **f** Expression of naïve, effector, memory, cytotoxic and regulatory markers across different CD8 T cell populations.

To further explore baseline immune correlates of response to JNJ-0535, scRNA-seq analysis on whole blood was performed. Unfortunately, due to the complexity of the sampling and analysis procedure, scRNA-seq data was only generated in the HV cohort and not in the CHB study. Because the analysis was performed on red-blood-cell-depleted, freshly collected whole blood, this dataset consists of a deep peripheral immune profile in the HV cohort, including neutrophils, which are typically lost after PBMC isolation and cryopreservation (Fig. 4a). The HV cohort was divided in response groups, based on the maximum ELISPOT response: (A) High response group ( ≥ 500 SFU/10^6^; n = 3); (B) Mid response group (200 – 500 SFU/10^6^; n = 3); (C) Low response group (23.8 – 200 SFU/10^6^; n = 5); (D) No response group ( < 23.8 SFU/10^6^; n = 1). First, the abundance of CD4 and CD8 T cell subsets in baseline samples across response groups was compared. Response group D was clearly different from all the other groups, having lower abundance of naïve CD4 and CD8 T cell subsets and higher abundance of effector CD4 (CD4_GATA3) and CD8 (CD8_GZMK) T cell subsets (Fig. 4b, c). The ratio of CD4 naïve-to-CD4 GATA3 and CD8 naïve-to-CD8_GZMK was lower in group D compared to the other groups (e.g., CD4 naïve-to-CD4 GATA3: geometric mean Group A = 8.6 vs Group D = 1.9) (Fig. 4d). CD4_GATA3 had high expression of both memory (*IL7R*) and effector (*GATA3*) markers, but low expression of naïve markers (*TCF7, LEF1, CCR7*) (Fig. 4e). CD8_GZMK had high expression of effector (*GZMK, KLRB1, CCL5*), exhaustion (*TIGIT*) and memory (*IL7R*) markers, but low expression of cytotoxic (*GZMB, KLRD1*) and naïve (*CCR7, LEF1, TCF7*) markers (Fig. 4f).

### Vaccine-induced CD8 T-cell responses are associated with baseline type-1-interferon-stimulated neutrophils/monocytes in HVs

**Fig. 5:**
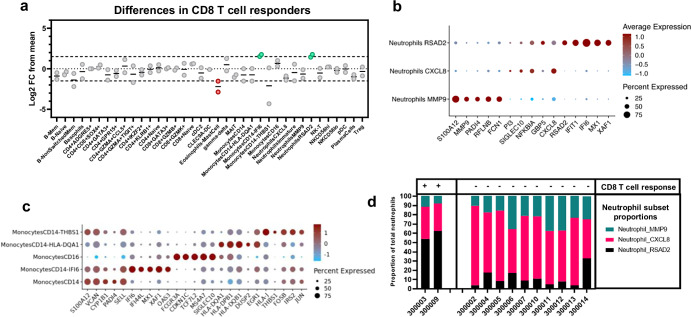
HVs mounting vaccine-induced CD8 T cells have higher type 1 interferon stimulated neutrophils and monocytes at baseline. **a** Log2 fold change (vs mean) of proportion of all whole blood immune cell populations of 2 HVs with CD8 T cell responses post-vaccination. **b** Dot plot with key expression markers of different neutrophil subsets. **c** Dot plot with key expression markers of different monocyte subsets. **d** Proportions of different neutrophil subsets in HVs mounting CD8 T cell responders vs non-responders.

Although 11/12 HVs mounted a significant IFN-γ ELISPOT response, ICS analysis demonstrated that only 2/12 HVs mounted polyfunctional CD8 T cell responses in addition to CD4 T cell responses (Fig. 2). To explore baseline immune correlates associated with vaccine-induced CD8 T-cell responses, the scRNA-seq proportions of all immune subsets from the two CD8 T cell responders were compared against the mean of the proportion of all immune subsets from non-responders (Fig. 5a). Three immune cell populations were different in both CD8 T cell responders compared to the rest of the participants: (1) Lower abundance of eosinophils/mast cells; (2) Higher abundance of Monocytes_IFI6 and (3) Higher abundance of Neutrophils_RSAD2 (Fig. 5a). Neutrophils_RSAD2 had a type 1 interferon stimulated gene expression profile, expressing typical interferon stimulated genes (ISGs) such as *RSAD2, IFIT1, IFI6, MX1 and XAF1* (Fig. 5b). Similarly, Monocytes_IFI6 showed a comparable type 1 interferon stimulated gene expression profile, expressing typical ISGs such as *IFI6, IFI44L, MX1, XAF1* and *OAS3* (Fig. 5c). Comparison of the proportion of different neutrophils subsets between CD8 T cell responders and non-responders clearly showed higher proportion of type 1 interferon-stimulated neutrophils (Neutrophils_RSAD2) in the response group, suggesting that myeloid cells (monocytes/neutrophils) with an ISG expression signature should be further explored as baseline biomarker for vaccine induced CD8 T cell responses.

## Discussion

JNJ-0535, a DNA vaccine encoding HBV antigens core and pol, was developed for treatment of CHB. To enhance immunogenicity of the vaccine, JNJ-0535 was administered using an electroporation device named TDS-IM v2.0. Electroporation enhances intracellular uptake of vaccines, showing a significant enhancement of the potency of DNA vaccines compared with conventional i.m. injection^10^. 3 injections of JNJ-0535 with electroporation were assessed in 2 clinical studies, a first-in-human, phase I, dose-escalation study in patients with CHB and a single-dose study in HVs.

The overall safety of JNJ-0535 was similar in HVs and CHB patients, and AEs were limited to local reactions at the injection site. In addition, we observed no evidence of ALT flares and viral breakthroughs in vaccinated patients. However, no relevant changes in HBsAg levels and other viral parameters were observed and no patient had HBsAg loss or seroconversion during the study. This confirms other clinical trials used for therapeutic vaccination^11,12^. The lack of efficacy in the CHB cohort is likely multifaceted: (1) JNJ-0535 was not able to generate and maintain polyfunctional CD4 and CD8 T cells, needed for successful immunity against HBV, (2) the chronic exposure to high levels of viral antigens (mainly HBsAg) might render liver-homing HBV-specific T cells dysfunctional, preventing clearance of infected hepatocytes. While vaccination elicited durable HBV-specific T-cell responses in HVs, characterized by high responder rates, responses in CHB patients were attenuated. Most vaccine responses in CHB patients were transient, and the frequency of responders decreased during follow-up, suggesting a potential impairment in eliciting durable cellular immunity in this population. Both studies did not sample liver, nor were we able to further explore HBV-specific T cell phenotype due to the limitation in PBMC samples and the requirement to use these samples to support secondary objectives (ELISPOT, ICS). In a follow-up study in HBeAg-negative virologically suppressed CHB patients, we are exploring these questions more in depth (OSPREY), but this is not in scope for this publication. The absence of expansion or lack off polyfunctionality of core-specific CD8 T cells in CHB patients after vaccination, could reflect the state of HBV-specific CD8 T-cell exhaustion and dysfunction that is known to occur in chronic HBV infection. Pre-existing exhausted CD8 T cells in chronic infections express inhibitory receptors, such as PD-1, exhibit diminished proliferative capacity, reduced cytokine production, and impaired cytotoxic function, factors that could collectively limit their response to the vaccine. The fact that some CHB patients demonstrated increases in IFN-γ secretion without polyfunctionality might suggest partial re-activation after vaccination. In addition, stimulation by antigen presenting cells, CD4 helper T cells, or other innate immune cells, and inhibitory signals can contribute to the abnormal priming of naïve CD8 T cells after vaccination. To further elucidate the rarity of CD8 + T cell responses in CHB patients, additional mechanistic studies assessing T cell exhaustion phenotypes and liver-specific T cell trafficking will be necessary to address this limitation. It is important to interpret these findings cautiously, given the small number of participants in both studies.

The lower immunogenicity to JNJ-0535 in the CHB cohort is likely not related to the vaccine design or administration route, as JNJ-0535 induced a durable HBV-specific T-cell response towards both core and pol antigens in the majority of HVs. Notably, the only HV that did not mount a T cell response to JNJ-0535 presented with a distinct overall T cell immune profile at baseline, characterized by a low ratio of naïve over effector/memory T cells (both CD4 and CD8). The homologous prime-boost vaccination strategy in this study is designed to prime naïve antigen-specific T cells during the first dose and boosting memory antigen-specific T cells during the following doses. A general reduction of the naïve T cell pool might thus impact T cell responses in this vaccination regimen. High frequencies of pre-existing memory and low proportion of naïve T cells have been shown to be predictive of poor T cell responses in SARS-CoV-2 vaccination studies, and this immunotype is found in the elderly and is related to, persistent antigen stimulation and an inflammatory environment^13,14^. Persistent antigen stimulation in chronic HBV infection might thus mimic this immunotype in an antigen-specific manner, leading to depletion of the naïve HBV-specific and poor T cell responses during prime-boost vaccination. Indeed, tetramer-based HBV-specific T cell analyses have shown low abundance of naïve and a high abundance of antigen-experienced memory core- and polymerase specific T cells in CHB patients^15,16^. In a setting of chronic antigen stimulation, the persistence of a rare population of stem-like T cells (associated with expression of the transcription factor TCF1) is essential to induce highly functional effector T cells following vaccination or immune checkpoint inhibition therapy^17^. Whether these stem-like HBV-specific T cells are present in peripheral lymph nodes, or enriched in the liver micro-environment, remains to be determined and will influence future therapeutic vaccination strategies in CHB, such as systemic vaccination and/or combination regimens to promote proliferation of this T cell subset.

Our data also support the importance of innate immunity in the generation of vaccine-induced CD8 T cell responses, in particular type 1 interferon signaling in innate immune cells. We indeed show that neutrophils and monocytes with an ISG expression signature are abundant in HVs mounting CD8 T cell responses. Type 1 interferons (IFN-α/β) are well known for their role in driving CD8 T cell immunity by regulating activation and increased antigen presentation of cross-presenting cells, mainly type 1 classical dendritic cells (cDC1)^18–20^. Type 1 interferons can also shape CD8 T cell immunity through other innate immune cells, as it was recently shown that type 1 interferon-stimulated neutrophils not only act as a biomarker of response to checkpoint inhibition in cancer but also function as an immunomodulator creating an environment permissive to CD8 T cell activation through secretion of known activating factors such as IL-12b. Taken together, these data provide additional support for adjuvants as indispensable components of therapeutic vaccines, in particular adjuvating components that boost the type 1 interferon response.

The small size and uncertainties with translatability of findings in a healthy to a chronically infected setting limit definitive conclusion. Our findings may suggest that an altered innate immune cell status, T-cell exhaustion and reduced naïve T cell pools play a role in vaccine responsiveness. Enhancing innate immune activation (e.g., via adjuvants that stimulate IFN pathways such as TLR7, 8 and 9) or reversing T-cell exhaustion (e.g., through checkpoint blockade such as anti-PD1 and/or lowering viral antigens through small-interfering RNA (siRNA)) could improve vaccine efficacy. It is possible that the selected study population influenced the outcome of the CHB study and that participants at earlier phases of the disease (i.e. HBeAg positive) would have shown different responses to the vaccination since their HBV specific T cells are described as less dysfunctional. Moreover, combination strategies that incorporate vaccination with these immunomodulatory approaches should be considered as promising avenues to optimize treatment outcomes in CHB patients. Future studies should include larger cohorts with comprehensive immune profiling to validate these potential biomarkers identified in HV and elucidate mechanisms underlying vaccine non-responsiveness in CHB. Addressing these immune barriers will be critical for the development of effective HBV immunotherapies. The follow-up study, OSPREY, was initiated to evaluate whether the lowering of HBsAg with the siRNA (JNJ-73763989) prior to vaccination with JNJ-0535 would boost HBV-specific immune responses. The results from this study may further inform if these immunogenic effects can lead to clinical benefits for patients with CHB.

## Methods

### Compound

The DNA vaccine JNJ-64300535 (JNJ-0535) encodes HBV antigens core and pol, mixed in equal quantities by weight and administered i.m. via electroporation using the TDS-IM developed by Ichor. HBV core and pol aa sequences were aligned in silico to generate a consensus sequence for the HBV B, C, and D genotypes. When the adapted BCD consensus sequence was compared to an A consensus sequence, 99% and 95% similarity was observed for core and pol, respectively. JNJ-0535 was manufactured under current good manufacturing practice guidelines.

### Clinical study designs

64300535HPB1001 (NCT03463369) is a double-blind, randomized, placebo-controlled, first-in-human, Phase 1 study to evaluate the safety, tolerability, reactogenicity, and immunogenicity of multiple, ascending doses of the novel HBV DNA-based, therapeutic vaccine JNJ-0535 administered by electroporation-mediated i.m. injection using a novel delivery device (i.e., TDS-IM v2.0). Study initiation date was 18 April 2018, and the study completion date was 23 March 2021. Individual study duration was approximately 66 weeks (a screening phase of 6 weeks, a vaccination phase of 12 weeks, and a follow-up phase of 46 weeks). The study was conducted in Belgium, Germany, and the UK and enrolled patients with hepatitis B e antigen (HBeAg)-negative, virologically suppressed CHB patients receiving stable NA treatment for ≥12 months. Eligible patients were both male and female, 18 to 65 years old with normal alanine transaminase (ALT), HBV DNA < 60 IU/mL and HBsAg of 100 to 10,000 IU/mL. A central interactive voice/web response system was used for randomization and to assign treatment codes and matching study kits to participants. JNJ-0535 was administered on Day 1, Week 4, and Week 12 in three different dose levels in cohorts A, B, and C with a low dose of 0.25 mg of plasmid (i.e., 0.125 mg + 0.125 mg of each plasmid) : placebo (n = 6:2), a medium dose of 1 mg of plasmid : placebo (n = 6:2), and the maximally feasible dose (MFD) of 6 mg of plasmid : placebo (n = 10:3) per injection, respectively. Each dose level cohort was expanded after a safety assessment of a sentinel group with 1 patient each on active and placebo. Dose escalation was governed by the sponsor data review committee (DRC) based on unblinded assessment of safety and immunogenicity data. Participants, site staff and Sponsor remained blinded until study completion.

Primary endpoints included the incidence and severity of adverse events (AEs), laboratory abnormalities, vital signs, and reactogenicity (up to 7 days post-last dose). Secondary endpoints encompassed HBV-specific T-cell responses assessed by IFN-γ ELISpot, response timing, cytokine profiles of CD4+ and CD8 + T cells via Intracellular Cytokine Staining (ICS), and device fault observations during treatment.

64300535HPB1003 (NCT04736147) is a Phase 1 single-arm, open-label study to assess the immunogenicity, safety, and reactogenicity of JNJ-0535 in HBsAg-negative HVs. Study initiation date was 3 February 2021, and the study completion date was 7 December 2021. Individual study duration was approximately 36 weeks (a screening phase of maximum 4 weeks, a vaccination phase of 8 weeks, and a follow-up phase of 24 weeks). All participants, healthy adult males aged 18 to 55 years, received the 6 mg dose administered at Day 1, Week 4, and Week 8. HV without a history of HBV infection were enrolled at a single center in Belgium and, and all participants were HBsAg and anti-HBc negative at the time of enrollment. HV who had received prophylactic HBV vaccination were not excluded, and data regarding prior vaccination were not collected, as these vaccines contain surface antigens, which are not included among the antigens targeted by JNJ-0535.

No formal sample size calculation was performed for either of the exploratory studies. The number of participants selected was deemed adequate for a preliminary assessment of immunogenicity and safety.

Both studies were conducted according to ethical principles that have their origin in the Declaration of Helsinki and are consistent with good clinical practice. Study protocols were approved by the local independent ethics committee/institutional review board. Written informed consent was obtained from all study patients.

### Study procedures

Safety and tolerability of JNJ-0535 were evaluated by assessments for adverse events (AEs), abnormal clinical laboratory tests, 12-lead electrocardiogram, vital signs, and physical examination.

Virology and serology parameters including HBV DNA, HBV RNA, HBsAg, and hepatitis B core-related antigen (HBcrAg) were centrally assessed in CHB patients prior, during, and after JNJ-0535 vaccination. HBV DNA viral breakthrough was defined as having a confirmed on-treatment HBV DNA increase by >2 log_10_ IU/mL from nadir level or confirmed on-treatment HBV DNA level >1000 IU/mL in participants who previously had HBV DNA levels below the lower limit of quantification (LLOQ) of the assay.

Peripheral blood mononuclear cells (PBMCs) were isolated from whole blood obtained from HVs and CHB patients. For this, venous blood was collected in lithium heparin-coated blood collection tubes prior to and at 10 or 14 days post each vaccination and at several timepoints during follow-up (FU).

PBMCs were isolated according to the standardized procedure for PBMC isolation and cryopreservation. In brief, venous blood samples were diluted 1:2 in Hanks buffered salt solution (HBSS). PBMCs were isolated by isopycnic centrifugation using Lymphoprep™. Subsequently, cells were washed twice in HBSS, suspended in freezing medium (i.e., 10% dimethylsulfoxide (DMSO)/90% fetal bovine serum [FBS]), frozen at a concentration of 10 million cells/mL within 10 hours after blood collection and finally stored in liquid nitrogen until use.

### ELISpot detection of HBV-specific T-cell responses

PBMCs isolated from participants were stimulated overnight with peptide pools of 15 amino acids overlapping by 11 amino acids (JPT peptide Technologies) covering the entire HBV core (40 peptides) and pol antigens, designed to match the BCD consensus sequence of the vaccine inserts for both core and pol proteins.

Peptide pools for pol were split into 2 pools, i.e., pol1 (107 peptides) and pol2 (107 peptides), to cover the entire protein sequence. Internal controls were dimethyl sulfoxide (DMSO, unstimulated/back-ground control) and CEF-T (i.e., human leukocyte antigen [HLA]-I and II restricted peptides from cytomegalovirus, Epstein-Barr virus, influenza, and tetanus toxoid) as positive control. Each condition was tested in triplicate at 2 ×105 PBMC/well. Peptide-pool-positive results were background (DMSO) subtracted and mean responses were reported. The IFN-γ ELISpot assay was performed using the human IFN-γ ELISpot PRO kit (3420-2APW-2, Mabtech). Spots were enumerated using an automated spot counter (ImmunoSpot^®^ S5, Cellular Technology Limited).

To determine a threshold of positivity (PT), T-cell DMSO responses to each individual peptide pool were analyzed in 40 HBV-naïve healthy donors, subsequently the 97,5th percentile was calculated for each individual peptide pool on log-transformed values. PT was observed at 11.3, 50.9, and 11.9 SFU/million PBMC for respectively HBV pol1, HBV pol2, and HBV core.

A baseline detectable response was defined as a value (spot-forming units [SFU]/million PBMCs) ≥ PT for a specific peptide pool prior to vaccination. Values < PT/2 are imputed by PT/2.

For the study in HVs, a response to the vaccine was defined as ≥3-fold increase compared to the PT. If a participant already had an ELISpot response value above the PT at baseline, a response was defined as a 3-fold increase from baseline. A responder was defined as a participant with a response against any vaccine antigen (core and/or pol) observed at 2 timepoints or 10 days after the last vaccination.

For the pooled analysis comparing HVs and CHB patients, a response post-vaccination was defined as a value (SFU/million PBMCs) ≥ PT for a specific peptide pool showing a ≥ 3-fold increase compared to baseline. A responder was defined as a participant with a response at any timepoint post-vaccination against any of the antigens (core, pol) included in the vaccine.

### Intracellular cytokine detection of HBV-specific T-cell responses

PBMCs at 2 ×106 PBMC/well were stimulated overnight with the same peptide pools and internal controls as used for the ELISpot assay described above. Cells were stained with VIVID (ref. L34957 LIVE/DEATH^tm^ fixable dead cell stain kit, Invitrogen), CD4-Peridinin Chlorophyll (ref. 345770, BD Biosciences), CD8-AlloPhycoCyanin H7 (ref. 641400, BD Biosciences), fixed and permeabilized (Cytofix/Cytoperm kit, BD Biosciences) with CD3-AlexaFluor 700 (ref. 557943, BD Biosciences), IFN-γ-fluorescein isothiocyanate (ref. 554551, BD Biosciences), interleukin (IL)-2-allophycocyanin (ref. 554567, BD Biosciences), CD154-phycoerytrine (ref. 555700, BD Biosciences), tumor necrosis factor (TNF)-α-phycoerytrine-cyanine7 (ref. 557647, BD Biosciences). Flow cytometry was performed on a BD LSR FORTESSA X-20.

### Olink^®^ Explore 3072 serum profiling

Serum was collected at baseline in both HVs (6430535HPB1003, n = 11, active 6 mg treatment group) and CHB patients (6430535HPB1001, n = 15; n = 9 in the active 6 mg treatment group and n = 6 pooled placebo group) in serum separation tubes (BD Vacutainer® SST tubes) and cryopreserved in 2 mL cryovials. Proteins were measured in thawed serum using Olink^®^ Explore 3072 (Olink Proteomics AB, Uppsala, Sweden) according to the manufacturer’s instructions. Supervised differential protein abundance analysis was performed comparing CHB versus HV.

The protein expression levels that showed differential abundance were also compared between vaccine responders (n = 5) and non-responders (n = 4) among CHB patients in the active treatment group receiving 6 mg of JNJ-0535 (n = 9).

### Single-cell RNA-sequencing and data analysis

Whole blood was collected in a 1.1 mL Lithium Heparin Sarstedt S-Monovette and shipped immediately to the processing laboratory. Within 6 hours of blood collection, red blood cells were depleted with EasySep™ RBC depletion reagent (StemCell Technologies). Cells were manually counted, and 20,000 cells were loaded on the BD Rhapsody Express Unit. Single cells were isolated using single-cell capture and cDNA synthesis with the BD Rhapsody Express Single-Cell Analysis System according to the manufacturer’s recommendations (BD Biosciences). cDNA libraries were prepared using the BD Rhapsody Whole Transcriptome Analysis Amplification Kit following the BD Rhapsody System mRNA Whole Transcriptome Analysis (WTA). The final libraries were quantified using a Qubit Fluorometer with the Qubit dsDNA HS Kit (ThermoFisher) and the size distribution was measured using the Agilent high-sensitivity D5000 assay on a TapeStation 4200 system (Agilent technologies). Sequencing was performed in paired-end mode (2× 75 cycles) on a NovaSeq 6000 (Illumina) using a NovaSeq 6000 S2 Reagent Kit (200 cycles). Cells with <500 unique molecular identifiers (UMIs) or <250 genes or >25% mitochondrial counts were removed from downstream analysis. All downstream analysis was done in R using the Seurat v4.1.1 package. Data was log-normalized with a scaling factor of 10,000. For the first-level clustering, the top 2000 most variable genes were selected (‘vst’ method implemented in FindVariableFeatures) and scaled using a linear model in the ScaleData function. Afterwards, principal component analysis (PCA) was run, the number of significant principal components (PCs) to be used for downstream cell clustering was determined using Jackstraw and heatmap plot inspection. A nearest neighbor graph and uniform manifold approximation and projection (UMAP) plot were generated using the significant PCs. A Louvain clustering was run on all cells, and the best resolution for clustering was determined using an average silhouette scoring across all clusters, testing 10 resolutions between 0.1 and 1 as previously implemented in Ziegler et al. (22). Marker genes for each cluster were calculated using the FindAllMarkers function (method= ‘wilcox’) implemented in Seurat and each cluster was iteratively subclustered further using the same approach. Subclustering was stopped when the resulting clusters were not meaningfully different. Clusters were annotated as cell type populations based on the expression of genes that are known markers of specific cells by expert annotation.

### Statistical analysis

The number of participants selected for this study was deemed adequate for a preliminary assessment of safety and immunogenicity. No formal hypothesis testing was done. Safety (i.e., labs, vital signs, electrocardiogram) and immunogenicity were analyzed descriptively by treatment for all participants who received at least 1 vaccination. Treatment-emergent adverse events (TEAEs) are AEs with onset after first vaccination or that worsened since baseline and were coded in accordance with the Medical Dictionary for Regulatory Activities (MedDRA), Version 20.1.

For the immunogenicity analysis, mock -subtracted values were evaluated at baseline and at different time points during the vaccination period and during the FU period ( ≥ 12 weeks after the last vaccination) as follows. A detectable baseline response was defined as a mock-subtracted value (spot-forming cells [SFU]/million PBMC) ≥ PT for a specific peptide pool prior to vaccination. A response during vaccination or FU for a specific peptide pool was defined as a mock-subtracted value (SFU/million PBMC) ≥ PT showing a ≥ 3-fold increase compared to baseline. A responder for a specific peptide pool was defined as a participant with a response at any timepoint during the vaccination period, against that specific peptide pool. A responder was defined as a participant with a response at any timepoint during the vaccination period, against any of the antigens (core, pol) included in the vaccine.

## Supplementary information

NPJ vaccines supplementary data

## Data Availability

The data sharing policy of Janssen Pharmaceutical Companies of Johnson & Johnson is available at https://www.janssen.com/clinicaltrials/transparency. As noted on this site, requests for access to the study data can be submitted through Yale Open Data Access (YODA) Project site at http://yoda.yale.edu. The single-cell RNA sequencing dataset generated and analyzed during the current study is available in the Zenodo repository https://zenodo.org/records/17120218. The Proteomics dataset generated and analyzed during the current study is available in the Zenodo repository https://zenodo.org/records/17292246.
